# Scientific Production about the Adherence to Antiretroviral Therapy

**DOI:** 10.3823/2514

**Published:** 2017-09-10

**Authors:** Regina Célia de Oliveira, Danielle Chianca de Andrade Moraes, Cleytiane Stephany Silva Santos, Gicely Regina Sobral da Silva Monteiro, Juliana da Rocha Cabral, Roberta Andrade Beltrão, Calos Roberto Lyra da Silva

**Affiliations:** 1 Nurse. Nursing doctor. Assistant professor at Nossa Senhoras das Graças Pernambuco University. Vice-coordinator at post graduation associated program UPE/UEPB, Recife, Pernambuco, Brazil; 2 Nurse. Nursing PhD student by the post graduation associated program UPE/UEPB, Recife, Pernambuco, Brazil; 3 Nursing bachelor degree academic at Nossa Senhora das Graças Pernambuco University, Recife, Pernambuco, Brazil; 4 Nurse. Nursing master degree by the post graduation associated program UPE/UEPB, Recife, Pernambuco, Brazil; 5 Nurse. Nursing doctor. Assistant professor at the Master’s degree program at Alfredo Pinto Nursery School from UNIRIO, Rio de Janeiro, Rio de Janeiro, Brazil

**Keywords:** Antiretroviral Therapy, Highly Active, Medication Adherence, Bibliometric, HIV, Acquired Immunodeficiency Syndrome

## Abstract

**Objective:**

To identify the elite of authors about the subject adherence to antiretroviral therapy; to identify the journals turned to publishing articles about adherence to antiretroviral therapy; and to identify and analyze the most commonly used words in abstracts of articles about adherence to antiretroviral therapy.

**Method:**

A bibliometric study conducted through the Scopus base. We used articles published between 1996 and 2014, after application of the eligibility criteria, there were composed the sample with 24 articles. The data were analyzed descriptively. Were used the laws of bibliometric (Lotka, Bradford and Zipf) and the conceptual cloud map of words, through the program Cmap tools.

**Results:**

Lotka’s Law identified the 5 authors more productive (46% of the total published). Bradford is impaired in this study. Concerning Zipf, 3 zones were determined, 31.47% of the words with in the first zone, 26.46% in the second and 42.06% in the third. In the conceptual map, the words/factors that positively and negatively influence adherence were emphasized, among them the need for more research in the health services.

**Conclusion:**

There are few publications about the accession to antiretroviral therapy, and the scientific production is in the process of maturation. One can infer that the theme researched is not yet an obsolete topic. It should be noted that the Bibliometric was a relevant statistic tool to generate information about the publications about the antiretroviral therapy.

## Introduction

Approximately 36.7 millions of people lived with HIV/AIDS in the world, till the end of 2015 [1]. In Brazil, were notified till June of 2015, 798,366 people with HIV/AIDS; from those, about 450 thousand used the antiretroviral therapy [2]. Even with an increasing of notification of heterosexual infected women, the recent studies points that the larger prevalence of HIV is still on the male gender, heterosexuals and singles. The more prevalent age group is from 30 to 49 years old, characterized for being an economically active population [2–4];

Starting from the law 9.313/1996, Brazil passed to guarantee free distribution of the TARV to all the infected by HIV (*Human Immunodeficiency Virus)* and who have the medical indication to receive it [5]. Since then, the AIDS Brazilian program is presenting important health indicators, as the morbid mortalities what reflects to improve of life quality to whom live with HIV/AIDS and adhere to the treatment [6].

The TARV objectives the reduction of the viral charge at the PVHA till turns it undetectable. However, to that can occur, it is necessary the faithful adhesion to the treatment. The incorrect and discontinuous use of the antiretroviral can result in resistance due to viral mutations that make the patient not answer to the treatment in a satisfactory way [7].

The collateral effects are the main causers to the abandonment at the beginning of the treatment, bearing in mind that they can cause diarrhea, nausea, and vomits, among others. Still the prescribed pills quantities, the caring with some antiretroviral, when they need refrigeration, the use of alcohol and others drugs and the difficulty to reconcile the medication taking with working activities are also factors that influence on the treatment adhesion [8].

That way, the TARV adhesion evolves not only the medication taking, but also socio economical and psychological factors, familiar and social support. It is highlighted that the precocious orientation to the treatment persistence. Favoring the adhesion to the TARV pass through the medical choose scheme till the routine adequacy and the number of daily pills. It is about a daily conquer and shared between the PVHA and the multi professional team responsible daily for their assistance, because they shall be attentive to guide properly and investigate the main factors related to the non adhesion. [4,9].

Given the above, glimpsing the importance of this thematic, emerged the interest to conduct this bibliometric research, from which emerged the following guidance question: which are the bibliometrical indicators of the scientific production about the adhesion to the antiretroviral therapy at the Scopus?

Thus this study has as objectives: to identify the elite of authors about the theme adhesion to the TARV; identify the journals devoted to the article publication about the adhesion to TARV; and identify and analyze the most common words at the articles abstracts about the TARV adhesion.

## Method

It is about a bibliometric study that allows us to analyze the data from the quantitative scientist articles and spread the information posteriorly [10]. This method use produce indicators that allow us to evaluate the results of the research with the finality to investigate the impact of the productions at the scientific community, through the statistic and *quantitative* data analyzes [11].

To elaborate this research, was conducted the following methodological route: theoretical foundation about the bibliometry and the adhesion to TARV aiming to scientifically base the study. Then we opted by choosing the Scopus data basis to the publications survey. This option emerged due to its relevance and international recognition at the scientifically community.

Were chosen the descriptions contained at the (MeSH): *Antiretroviral agents]*, *[Medication Adherence]* united by the Boolean operator AND, and posteriorly was made a simple research with the initials TARV (Adhesion to the antiretroviral therapy). All of them conducted at the same circumstances using the filter “article title”. “password” and “abstract”.

The temporal studied clipping was from 1996 to 2014. Were included on the study the documents recovered which the word “Adhesion to the antiretroviral therapy” consisted on the abstracts.

The documents recovering occurred in June 2015, being possible to recover 85 articles. From those, 63 were recovered at the first research from which 22 were included, whereas that at the second research, 22 were listed and only 2 included, totalizing thus a sample of 24 articles.

The data were tabled on Excel spreadsheets, generating thus the research data basis. The used bibliometric variables were: year of study publication, language, journal name, quality of the journal, impact factor of the journal and the authors, the institutions, considering only to the first author. Was applied the Bradford law to characterize the concentration places and the journal dispersion.

As for the key-words and abstracts, were applied the Lokta Law to identify, among other things, the authors elite [12]. The data were analyzed under the light of the descriptive statistic.

According to those laws, that are the principal bibliometric laws, in which the Zipf Law, there is a frequency of words at the scientific articles. By the Bradford law can be analyzed the journal productively, and through the Lotka Law, the scientific productively of the authors [12]. To help the Zpif application, the Ieamuteq software 07, alpha 2 were used to ranking and produce the cloud words. To the construction and analyzes of the conceptual map, was used the Cmap tools software.

To guarantee the methodological rigor and the reliability of the results that requires the bibliometric research, the selected data to the analysis were reviewed and collect by pairs. It wasn’t necessary the approval of the Ethics and research committee in human being to be about a bibliometric research.

## Results

The sample was composed by 24 articles, indexed from 1996 to 2014 on the Scopus basis.

**Figure 1:**
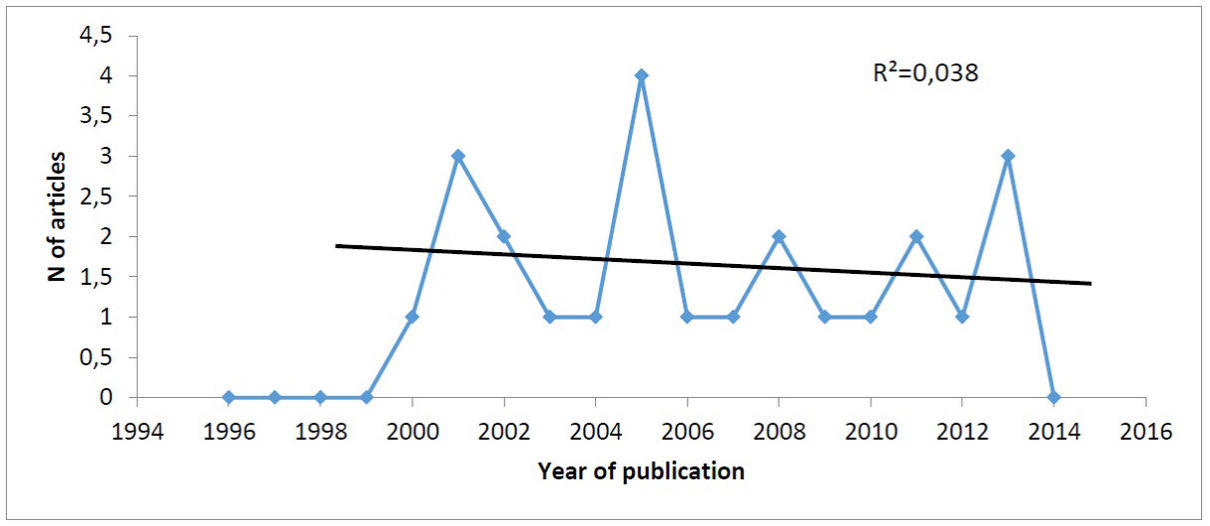
Frequency distribution of the scientific articles through the research for the descriptors contained at the *MeSH* [Antiretroviral agents] and [Medication Adherence], published between 2000 and 2013, on Scopus, Recife, PE, Brazil 2015. **Source:** Research data, 2015.

The Figure 1 evidences the distribution of the articles frequency by publication year and the quotient of determination (R^2^), which shows the behavior of the dispersion curve of the studies that approach the TARV thematic.

As for the scientific articles average, throughout the years, this resulted in 1.6; in relation to the variance, this was equal to 1.04 and the standard deviation of the population of 1.019804. It call our attention to the higher contributions from 2000 to 2002, 2004 to 2006 and 2012 to 2014.

Regarding the study caracterization, all were published in English. As for the kind of research, 41.7% were descriptives; 29.2% were literature review, 8.3% were controling-case, experimental and clipping, and only 4.2% didn’t inform at the abstract the kind of research. The majority was quantitative research (62.5%), only 4.2% had the qualitative approach and 33.3% were indetermined.

As for the authorship, was used complete authorship, it is, counted the author and the co-author, being the result 95 individuals. The estimated coefficient was of 4 authors per article, while the productively index was of 0.25, it is, less than an article by author. The authors and co-authors, besides the ranking and productively, were also classified according to the h index, which average was 13.6 varying from 1 to 92. It is noteworthy the low impact of Brazilian authors, not passing the h 2 index, while the North Americans represent the greater index h, 92.

When applying the Lotka Law, were achieved the following results: the total square root of the production is approximately 5, what represents that 5 more productive authors, together, contributed to 11 documents, representing approximately 46% of the total produce. This result approximates of the minimum of 50% of contribution on the scientific production, according to the elitism Law [13]. However, the results show difficulty to adequate the theoretical value of the elite to the square root criteria. Therefore, in this study, it was not possible to determine a group of elite to the studied theme.

**Figure 2:**
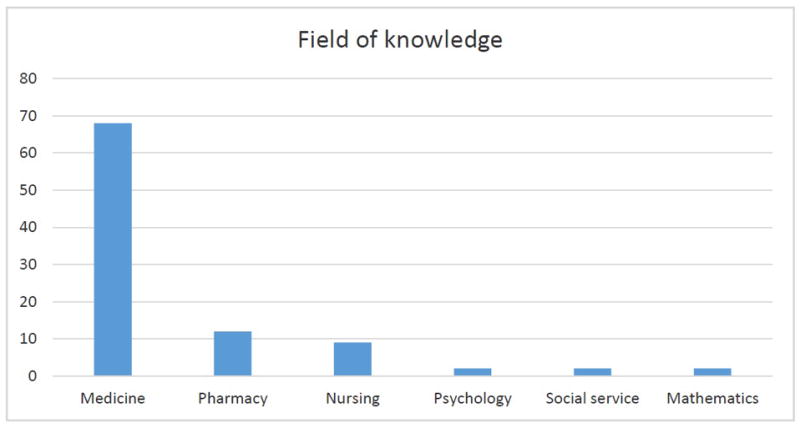
Distribution per knowledge area of the scientific articles through research by the descriptors contained at the *MeSH* [Antiretroviral agents] and [Medication Adherence], published from 2000 to 2013, on the Scopus basis, Recife, PE, Brazil 2015. **Source:** Research data, 2015.

The knowledge areas of the authors were also accounted and represented on the Figure 2, highlighting the major quantity to the medicine field (71.58%), followed by pharmacy (12.63%) and nursing (9.47%); the others areas together contributed with 6.32%.

As for the journals, were identifies 21 magazines or journals, classifies according to the qualis established by the CAPES (according to the instituted to the Nursing, when no informed, was searched for Medicine I or interdisciplinary), according to the Table 1.

After the application of the Bradford Law, in the Table 2, as concluded that this Law is prejudiced in this study, bearing in mind that only two magazines published more than one article; thereby, the ranking obeyed the higher index h.

As for the geo location, was identified that: in North America, the United States of America produced the majority of the articles (45.8%), while in South America, was verified (8.4%), in this continent, highlighting Brazil and Uruguay with 2.4% each one. In Africa, Togo contributed with 2.4%. The others (41.7%) didn’t inform their origin.

The lexicometrics analysis of the abstracts was obtained through the construction of a corpus that was processed on the Iramuteq software, using the UTF8 codification, according to the code **** *suj_1 till the **** *suj_24. After the data debugging, the final product was of 2.974 words, from which 47 occurred only once. The word of higher occurrence was [Adherence] with 122 occurrences.

When applying the first Law of Zipf (k=r.f). 3 zones were determined. In line with the theory, the two first zone should contain, each one, 991 words and the third, with 992. Empirically, achieved 936 words on the first zone (31.47%), 787 on the second (26.46%) and 1.251 on the third (42.06%). It was successively designed by the Zipf law as trivial or basic zone, interesting information, and for the last, noise.

The key-words table was ordered according to the frequency that the word occurred and its respective series order (r). Low order of the series corresponds to high words frequency, while the high order of the series corresponds to a low frequency, phenomenon designed by Zipf as the “less effort law”.

At the order of series containing two or more different words and with the same frequency, was conducted the pattern “mm”, obtained through the division of the sum of ® by the number of words correspondent to it. The zones are stablished according to the collor, being light gray zone 1, medium gray zone 2 and grey zone 3.

The distribution behavior of the words when applying the second Law of Zipf I_n_=I_1_x2/n (n=1). Where In is the occurrence frequency of the word, I_1_ the total of words occurred 1, and the constant 2 to any language. The results show a little difference between the theoretical and empiric values to the frequencies 2, 3, 4 and 5. In this way, the frequency 2 that should be 152 words was empirically of 164, the frequency 3 from 76 was of 76, the frequency 4 from 46 was 58, and the frequency 5 from 30, was computed empirically 34.

**Figure 3:**
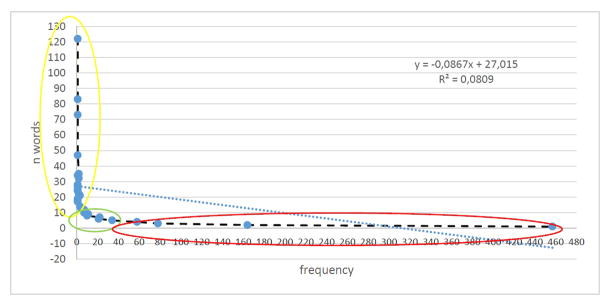
Zipf zone presented on the scientific articles through the research by the descriptors contained at the *MeSH* [Antiretroviral agents] and [Medication Adherence], published from 2000 to 2013, on the Scopus bases, Recife, Pe, Brazil, 2015. **Source:** Research data, 2015.

The transition point (T) was defined by the calculus of the formula proposed by Goffman. The result obtained was T=28 that, in turn, is located in the first zone, next to the board of the zone 2. This law describe the mathematical relation among the words, allowing classifying them as high and low frequency. The first law of Zipf shows that that, in some moments, there was a constant that proximate among the series orders. (r) Figure 3 [14].

**Figure 4:**
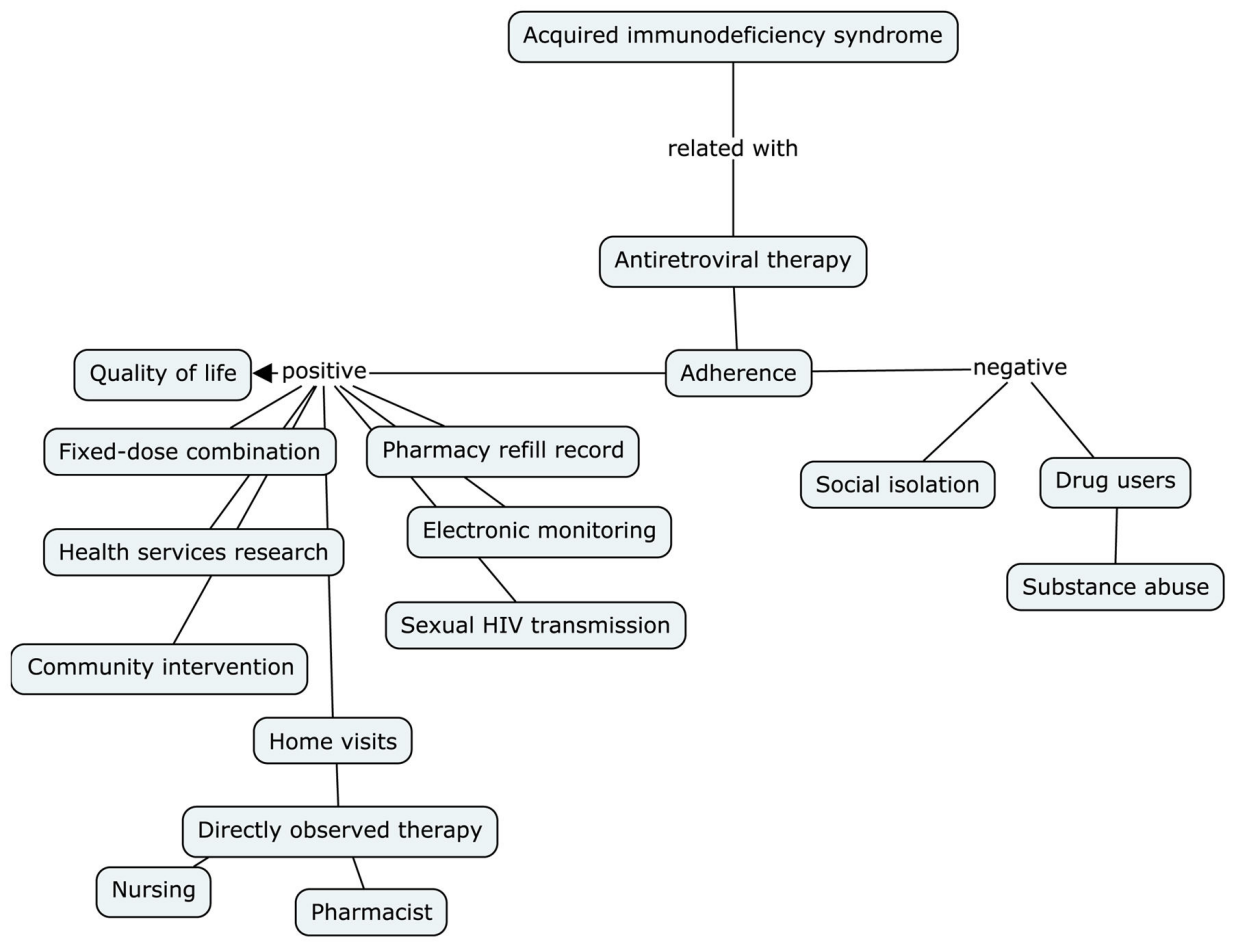
Conceptual map through the key-words presented on the research by the descriptors contained at the *MeSH* [Antiretroviral agents] and [Medication Adherence], published from 2000 to 2013, on the Scopus basis, Recife, PE, Brazil 2015. **Source:** Research data, 2015.

The Figure 4 presents the conceptual map through the key-words contained on the abstracts of the articles selected by the research, listing thus the factors that influence positively and negatively at the adhesion to the antiretroviral therapy.

It is observed that were considered factors that influences positively the adhesion to the TARV: Quality of life, Home visits, Pharmacy refill Record, the Electronic monitoring. About the factors that influence negatively the adhesion: Drugs user and Substance abuse, social isolation and the necessity to health services research.

## Discussion

In relation to the study publication year, the numbers present a discrepancy during the years. In this context, regarding the antiretroviral therapy, there was the consolidation of the therapeutic standard, from 1996 to 1999, starting from the advent of the new antiretroviral classes, which allowed originating the antiretroviral combined therapy [15]

This time, in the temporal clipping established to the study, it is observed that the publications began from 2000, what is passively related not only to the introduction of the TARV combined at the precedent years, but also at the necessary time space to elaboration and study conclusion evolving the thematic. This fact doesn’t prevent the existence of previous publications in 2000 in other data basis, or on the own used basis (Scopus) in a period before 1996.

Another relevant fact is that in 2002 was instituted the Global found to the combat against the AIDS, tuberculosis, and malaria, aiming to invest in resources to combat the epidemics from those grievances, including the extension to the access to TARV, providing thus, an increasing at the international consumption of those medications [16]. This political and social scenario influenced the actions to confront the HIV/AIDS, thus as is providing, maybe, more interest of the researchers in conducting studies about the adhesion to the TARV and, Posteriorly, occasioned an increase of publications, as it was possible to observe in 2005.

Referring to the determination coefficient (r^2^), was obtained the value equivalent to 0.0387. The oscillation between the publication year and the number of published articles shows a dispersion of the publication throughout the years. This allow us to infer that, although the TARV is a therapeutic modality of world reference at the HIV/AIDS treatment, more than 20 years, it is still published a little at the data basis researched regarding the adhesion to the antiretroviral. This is a concerning result, bearing in mind that it is necessary a continuous elevation to the TARV adhesion so that the viral charge levels can be kept undetectable [17].

Considering the minimum time 10 years to the maintenance of scientific production, this study shows that the production about the TARV is in maturation phase, still that weigh the facts the temporal clipping is superior to more than 10 years. It can be inferred that this theme is still found far from the obsolescence phase [18].

In reference of the study kind, 8.3% of the articles confers as control-case, experimental or clipping case. In this way, the literature points that the studies like this are excellent to the factors association evaluation, being its results of extreme relevance, but present as disadvantage the elevated cost to its conduction [19].

In the other hand, the descriptive studies were the most prevalent, and this fact can be related not only to the question of the low cost to the research conduction, but also enables tools of management much more important in health systems, being founded to inform about the distribution of an event in a population [19–20].

As for the methodological approach, the study with quantitative approach has the intention to guarantee a bigger precision, conducting to a result with few distortions possibilities, instead of the study with qualitative approach. In this line of reasoning, the problematic of the TARV adherence, was presented as being a research theme with larger necessity of precise results, seen that the majority of the articles found were researches with quantitative approach [21].

The results about the authorship of the articles reveals great quantity of authors and coauthors when compared to the article quantity, what demonstrates to exist a good distribution between the researchers and the articles being characterized so, in partnerships, even more probable by their participation in groups of multi central researches. Although the low productivity index (0.25), the qualitative aspect determined by the index h allows us to infer that the 24 articles manifest on the publication vehicle, so ever relatively shows a quantity little expressive. The journals which the authors published their articles, 33% are stratified in Qualis A1 and A2, while the journals B1 and B2 summed 30%.

Although the Lotka Law doesn’t determine in this study a elite group, bearing in mind that quantitatively the minimum percentage of 50of the total production is not produced by the 5 more productive authors, according to the determination of the square root of the total production law (Lotka law), the quality of those authors can be proved through the h index from which deserves to be highlighted that the average of this index among the five more productive authors is 50.5, allowing, thus, infer that the theme is being researched in renamed centers of research, thus, infer that the theme is being researched in renamed researched centers, by researchers of the best lineage. They are: Friedland, G.H. and An-dres, L.A.: Aids program, Yale University, School of Medicine, New Heaven, United States; Bova, C.A: Division of infectious Diseases, University of Massachusetts, Worcester, MA, United States; Davks KL: Department of Health Economics, RTI Health Solutions, Research Triangle Park, NC, United States and Dieckhaus, KD: Department of Infectious Diseases, University of Connecticut Health Center. Farmington, CT, United States.

As for the knowledge area, it was noticed that a prevalence of publication on the medicine area. This fact can be related to the therapeutic questions of the HIV/AIDS, seen that it is the doctor professional who is in front of the choice, use and collateral effects of the antiretroviral. However, to promote the integrality of the care in HIV/Aids it is essential that the multi professional team conduct interventions of adhesion tot the TARV, of an articulated way, and above all, with the doctors. In this way, it is reasonable infer that there is few interest by the others professionals who aren’t doctor in research about the TARV adherence, suggesting still that it is not homogenous the professional integration about this thematic [22].

The magazines where classified according to the most impact factor. The journal Clinical Infectious Diseases (CID) appear as the first of the series order of the Bradford table which metrics of the Scientific Journal Rankings – SJRe index h are, respectively, 4.34 and 247. This is stratified on the Qualis/CAPES as A1. Maybe what can justify this classification is the fact that a reference journal in publications on the infectious diseases area, that reaches a great international public with its theme about the clinical descriptions of the pathologies, infection prevention, evaluation of treatments used currently, therapeutic innovations, as well as the diagnostic founds and the treatments that are being conducted.

For being a really specific theme (TARV), the production counts with magazines highly specialized, as it is the case of the AIDS magazines, Journal of Acquired Immune Deficiency Syndromes, AIDS Reviews, AIDS and Behavior, Infectious Disease Clinic of North America, AIDS Care-Psychological and Socio-Medical Aspects of AIDS/HIV. Magazines that present also a high impact and, therefore, really desired, in its great majority by cutting-edge researchers, above all the one who produce clinical researches.

Regarding the institutions headquarters of the researches, the majority was in the United States of America – USA. This fact can be related to the historical-scientific aspects, in which the USA was the pioneer country on the HIV/AIDS discovery, as well as the studies evolving the antiretroviral. [23]

It is still noteworthy that although Africa presents the world greatest indicators of morbid mortality by aids [1], only a study was found in an African country. This fact can be related to the deficiency of investment from the own countries to the researches financing, besides the low HDI [24], configuring in a population with low perspectives of education and health.

The use of the Cmap tools program provided the construction of the conceptual map which facilitated the reorganization in a graphic and visual way of the key-words contained on the articles abstracts selected by the research, listing thus the factors that influence positively and negatively the antiretroviral therapy adherence.

Regarding the positive factors, it is remarkable the word quality of life, according to authors, the Family Health Unity is a space that aims to guarantee the health caring to the users and promote life quality [25], seen that the professionals are responsible for by the TARV treatment and essentials on the empowering of the patients searching for information in a critical and constructive way about their health status and ways to improve the life quality importance, in addiction to have to role to aware that the adherence is extremely important to a long life with more quality [26].

The association of the words: Home visits, Directly observed therapy, nursing and Pharmacist, are related to the importance of a multi professional assistance to the patients living with HIV/AIDS and with the actions that could improve the adhesion to the medication therapy, although there are professionals who says that the work overload and the reduced human resources on the health services hinders the home visits, preventing to guarantee a more effective following to those users.

We can also quote on the context positive factors to the adherence, the Pharmacy refill Record and the electronic monitoring, which are considered mechanisms of analyses of quantity and periodicity of the acquisition of the medications, besides being an alert to identify which patients present a potential risk to the non medication adherence. [25]

Those electronic registers besides verifying the irregular withdraw is possible to identify on the patients reports, the ones who complains and adverse events in relation to the treatment what can generate the therapy discontinuity. This following by the pharmaceutical allows us to observe which patients who continues with the initial scheme, or the ones who passed through changes on the first six months using, what hinders the adherence [25].

Another word found that deserves to be noticed was the sexual HIV transmission, because the knowledge about the transmission of the disease and the increase of the life expectative with the adequacy treatment, are factors that turns possible the health services to work not only in a preventive way, but also on the caring perspective respecting the singularity of the subject [27].

As for the factors that contributes to non adhesion of the antiretroviral therapy, was formed by a group with the words: Drugs user and Substance abuse, the finding of this research corroborates with the study conducted in Bahia that affirmed that the illicit drugs use is statistically related to the non adherence to the treatment [25].

Another word remarked on the key-words of the researched articles, was the social isolation, this can occur due to a failure on the patient support network, what corroborates with the finding the qualitative study conducted with 10 patients living with HIV/AIDS, in which 08 reveals the difficulty on the support network construction, which can be composed by family, partners and friends, this fragile bound is related to the stigma, fear of the prejudice and the difficult to accept the living conditions of those patients [27].

The social isolation is also referred during the medication taking moment, because due to the prejudice the patients who work aims to take the medication alone, due to the great quantity of medications throughout the day, what interferes to the daily life activities, besides causing the forgetfulness, facto that turns the adherence unfavorable [27].

On the actual conceptual map was verified also as support network to the community intervention and the fixed-dose combinations as possibilities to improve the medication adherence.

Giving the positive and negative factors that interfere on the TARV adherence, it is important to highlight the necessity of more Health services researches, aiming to spread the importance of the multi professional team and the familiar support network on the TARV adherence.

## Conclusion

Although that the therapy with antiretroviral is the world therapeutic model of choice to treatment of people living with HIV/AIDS, for almost two decades, it was observed that there are still few publications regarding the adherence to the anti-retroviral, besides the scientific production about this theme is in maturation phase, inferring that it is not about an obsolete theme, conducting, a certain concerning, bearing in mind the importance of the treatment adherence maintenance to the therapeutic success.

It is evident that the adherence to the TARV has an aspect considered multi factorial and it implies on the necessity to assistance with multi disciplinary view. However, it was noticed that there is few interest by the health professionals who aren’t doctors in research about the thematic, suggesting that, at least on the scientific area, there isn’t yet a homogeneity regarding this professional integration.

The theme is still being researched by renamed researching centers, as well as researchers from the highest lineage, in their majority, of North American origin. However, the adherence to the antiretroviral presented itself for being a research theme with more necessity of accurate results, bearing in mind the majority of the articles found have presented study design with a quantitative approach.

It was also verified that the necessity of studies that present as objects the health services to the promotion of the adherence to TARV, and also the focus on the importance of the multi professional team and the familiar support network.

It is noteworthy that the bibliometric was a statistic tool extremely relevant to generate information about the TARV publications. In relation to the authors who published about this thematic, the Lotka Law was efficient to list them. It was possible to identify the most used journals to the article publication about the TARV adherence through the Bradford law, finally, to identify the most used words on the articles abstracts, was applied the Zipf law.

Given the above, it is noteworthy the importance to conduct studies evolving the TARV adherence, as well as the financing aiming to deepen the aspects and technologies related to this thematic bearing in mind to provide ways to promote health and avoid grievances coming from a low treatment adherence.

## Figures and Tables

**Table 1:** Distribution of the scientific articles found on the research by the descriptors contained on the *MeSH* [Antiretroviral agents] and [Medication Adherence], published from 2000 to 2013, on the Scopus basis – Recife, PE, Brazil, 2015.

Ranking	Magazine	F	Σ partial	h-index	Qualis	SJR****
1°	Clinical Infectious Diseases	1	1	247	A1**	4.34
2°	AIDS	1	2	181	A1**	2.89
3°	Journal of Acquired Immune Deficiency Syndromes	3	5	120	A1*	2.22
7°	Pharmacotherapy	1	9	82	A2**	0.81
9°	AIDS Care - Psychological and Socio-Medical Aspects of AIDS/HIV	1	11	70	NI	0.98
5°	AIDS and Behavior	1	7	69	B2***	1.74
6°	Infectious Disease Clinics of North America	1	8	69	A2**	0.96
8°	American Journal of Managed Care	1	10	66	C*	0.94
13°	Computational Statistics and Data Analysis	1	16	63	A2**	1.39
19°	BMC Infectious Diseases	1	22	59	A1*	1.3
14°	Journal of the National Medical Association	1	17	47	NI	0.41
4°	AIDS Reviews	1	6	45	A**	1.48
10°	Current HIV Research	1	12	38	B1**	0.78
15°	Journal of the American Academy of Nurse Practitioners	1	18	29	NI	0.36
20°	BMC Clinical Pharmacology	1	23	25	B1*	0.72
11°	BMJ Open	1	13	24	B1*	1.13
17°	AIDS Research and Therapy	1	20	24	B1**	0.78
12°	Patient Preference and Adherence	2	15	17	B1*	0.59
16°	Revista chilena de infectología	1	19	13	B3**	0.21
18°	Journal of Pharmacy Practice	1	21	13	B3**	0.45
21°	Saúde e Sociedade	1	24	9	B1*	0.34

Source: Research data, 2015

* Qualis nursing;

** Qualis Medicine I;

*** Qualis multi professional; NI= non informed; Z=Zone, F= frequency, E= cumulative some.

**** ScientificJournalRankings –SJR

**Table 2:** Order of series and occurrence of words represented on the scientific articles through the research by the descriptors contained at the *MeSH* [Antiretroviral agents] and [Medication Adherence], published from 2000 to 2013, on the Scopus basis – Recife, PE, Brazil 2015.

Series order	N word	Frequency	f.n words	Σ partial	K=R.f
1	1	122	122	122	122
2	1	83	83	205	166
3	1	73	73	278	219
4	1	47	47	325	188
5.5	2	35	70	395	192.5
7	1	34	34	429	238
8.5	2	32	64	493	272
10	1	28	28	521	280
11	1	27	27	548	297
12	1	25	25	573	300
13	1	24	24	597	312
14.5	2	22	44	641	319
17	3	21	63	704	357
19	1	19	19	723	361
20	1	18	18	741	360
21	1	17	17	758	357
23	3	14	42	800	322
26.5	4	13	52	852	344.5
32	7	12	84	936	384
39.5	8	11	88	1024	434.5
47	7	10	70	1094	470
56	11	9	99	1193	504
66.5	10	8	80	1273	532
82.5	22	7	154	1427	577.5
104	21	6	126	1553	624
132.5	34	5	170	1723	662.5
177.5	58	4	232	1955	710
245.5	78	3	234	2189	736,5
366.5	164	2	328	2517	733
677	457	1	457	2974	677

Source: research data, 2015.
